# Oral and Lower Extremity Ulcers as the Initial Presentation of Granulomatosis with Polyangiitis

**DOI:** 10.1155/2022/2737242

**Published:** 2022-08-31

**Authors:** Mohammed Omar Al Salihi, Bianca Dominguez, Viresh Mohanlal, S. J. Carlan

**Affiliations:** ^1^Department of Internal Medicine, Orlando Regional Healthcare, Orlando, Florida, USA; ^2^Division of Nephrology, Orlando Regional Healthcare, Orlando, Florida, USA; ^3^Division of Academic Affairs and Research, Orlando Regional Healthcare, Orlando, Florida, USA

## Abstract

**Background:**

Granulomatosis with polyangiitis (GPA) is a small vessel vasculitis characterized by lung and kidney involvement. It is typically a disease of white females and has a poor prognosis with the average life expectancy of 5 months for a patient without treatment. Oral and skin ulcers are considered to be rare presentations.

**Case:**

A 39-year-old black male presented to the hospital with oral and skin ulcers and was diagnosed with GPA based on the biopsies of both cutaneous lesions and kidney. He was started on rituximab with minimal improvement. Later he was admitted to the ICU and had plasmapheresis, and he gradually improved and was discharged home 8 days after admission.

**Conclusion:**

GPA is an aggressive vascular disorder resulting in possible organ system damage and failure. The role of the sickle cell trait in this patient is undefined, but this combination of gender, race, and presenting symptoms in GPA is extremely unusual.

## 1. Introduction

Granulomatosis with polyangiitis (GPA), formerly known as Wegener granulomatosis, is a small vessel vasculitis with systemic manifestations. This disease is characterized by upper and lower respiratory granulomatous inflammation with or without renal involvement [1]. It is part of antineutrophil cytoplasmic antibody (ANCA) associated small vessel vasculitis which includes Churg–Strauss syndrome, microscopic polyangiitis, and renal-limited vasculitis [2]. GPA is a rare disease with a prevalence of 3 cases per 100,000 in the United States [3]. The localized form of the disease is predominant in young females [4], peaking in the fourth and sixth decade of life [5, 6]. GPA can present as a generalized disease or limited disease. The generalized disease can involve any organ while the localized form is usually limited to lungs, eyes, or ears, but other organs can be involved including the heart. Generalized symptoms such as fever and malaise may be present. Pulmonary symptoms include cough, hemoptysis, and pleuritic chest pain. Renal involvement is usually late and is associated with a poor prognosis [7]. Oral mucosa lesions occur in 6–50% of patients [8, 9]. Currently, the diagnosis of GPA is based on clinical, serological, and histological findings.

The diagnosis is aided by the presence of serum antinuclear cytoplasmic antibody. In the generalized disease, ANCA can be seen in 80–95% of the patients, but in limited presentations, ANCA can be detected in a range of 60%–80%. If clinical suspicion is high with a negative ANCA test, obtaining a tissue sample for histopathology is required for definitive diagnosis [1]. In our case, we present a male who is seen in the emergency department with cutaneous lesions as the initial presentation of GPA. Our patient initially presented without pulmonary or renal involvement and was diagnosed with GPA following biopsies of oral and cutaneous lesions.

## 2. Case Presentation

**Figure 1:**
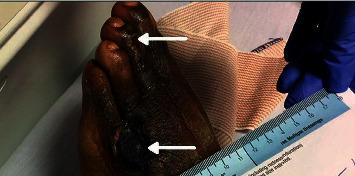
Ulcer on third and fifth toes (arrows).

A 39-year-old black male with known sickle cell trait presented to the emergency department with lower extremity swelling with blisters and odynophagia. He developed petechial lesions on both feet that progressed to ulcers (Figure 1) on the bilateral lower extremities. The odynophagia started 2 weeks prior to presentation and was associated with a tongue ulcer. He had been evaluated by an infectious disease team during a previous visit and had an unrevealing workup. He was not on any treatment for lower extremity lesions. He denied fever, chills, nausea, vomiting, diarrhea, weight loss, night sweats, recent travel, and history of IV drug use.

**Figure 2:**
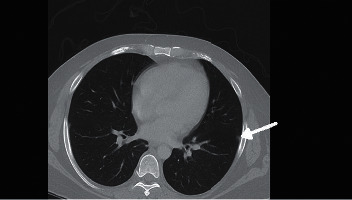
CT imaging showing multiple irregular pulmonary nodules (arrow) at different levels. The morphology of these nodules favors infectious/inflammatory nodules more than malignancy. Vasculitis can also have this appearance.

His initial vital signs were BP 156/86, HR 114, temperature 102.2 F, and O_2_ saturation at 98% on room air. Physical exam was remarkable for oral thrush with right tongue ulceration and healing ulcers on the dorsal surface of his feet bilaterally. His complete blood count (CBC) and basic metabolic profile (BMP) were within normal limits. Urinalysis was positive for blood and 2+ proteinuria. His urine toxicology was positive for oxycodone. He was initially treated for Systemic Inflammatory Response System (SIRS). Blood cultures were drawn and he was started empirically on vancomycin and piperacillin and tazobactam. Bilateral venous duplex was negative for deep vein thrombosis. Computed tomography (CT) of the chest showed multiple irregular nodules with suspected infectious versus inflammatory etiology and septic emboli could not be excluded (Figure 2). Transthoracic echocardiogram was negative for infective endocarditis. Bilateral foot ulcers drained serosanguinous fluid with cultures positive for *Enterococcus faecalis* and *Serratia* but four sets of blood cultures remained negative. Magnetic resonance imaging (MRI) of the left foot was normal and MRI of the right foot showed metatarsal stress reaction versus early osteomyelitis. Skin biopsy showed extensively necrotic fibroadipose tissue with acute inflammation. Right lateral oral tongue ulcerative lesion biopsy showed predominantly hyperplastic squamous mucosa showing an ulcer site and abscess with fragments of necrotic squamous epithelium associated with cytologic atypia and colonies of bacteria, and one fragment of necrotic squamous epithelium displayed vague papillary architecture. The patient later developed right eye conjunctivitis. Appropriate consultations with nephrology, pulmonology, and podiatry were obtained.

**Figure 3:**
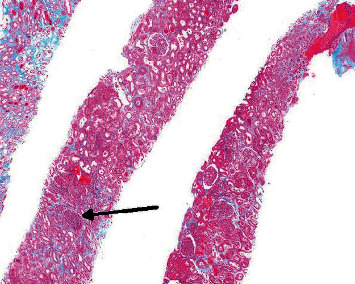
Light microscopy of a renal biopsy with multiple cores of renal tissue, of which approximately 40% is represented by cortex. Eighteen glomeruli are present, none of which are globally sclerotic. In up to four glomeruli, there are segmental cellular crescents, associated with fibrinoid necrosis and rupture of the capillary loops. Intact glomeruli do not show significant endocapillary hypercellularity. The tubulointerstitial compartment is marked by tubular injury, with tubular dilatation and epithelial simplification. There is mild and patchy inflammatory infiltrate, mixed. Multiple red blood cell casts are present throughout the biopsy. Occasional neutrophilic casts are also seen. There are mild interstitial fibrosis and tubular atrophy which comprise approximately 20% of the cortex. Arteries are marked by mild intimal fibrosis. Arterioles do not show significant hyalinosis. Toluidine blue-stained sections contain four intact glomeruli.

**Figure 4:**
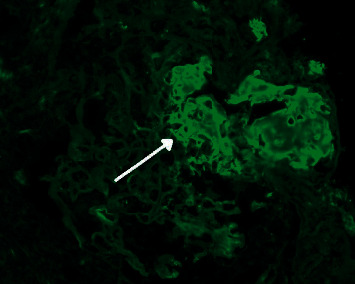
This section is stained for fibrinogen and kappa and lambda light chains. There is focal and segmental staining of glomeruli with fibrinogen, in areas of crescents/fibrinoid necrosis (arrow). Kappa and lambda stain equally throughout the tubulointerstitium.

**Figure 5:**
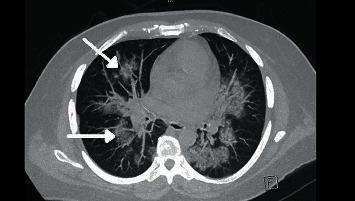
Predominantly central patchy and confluent ground-glass and interstitial opacities (arrows). This pattern is nonspecific but, given the patient's history, is favored to represent alveolar hemorrhage secondary to vasculitis.

Based on the constellation of oral and cutaneous ulcers, CT chest findings, bilateral foot ulcers, and conjunctivitis, a rheumatologic process was suspected. The antinuclear antibody (ANA) was negative, rheumatoid factor was with 1 : 640 titer, C-ANCA (antineutrophil cytoplasmic antibody) was positive with 1 : 1280 titer, and the diagnosis of Wegner's granulomatosis was made. He was started on rituximab 375 mg weekly and methylprednisolone 250 mg every 6 hours for 3 days. He later had a renal biopsy which showed granulomatosis with polyangiitis and mild interstitial fibrosis with tubular atrophy and a pauci immune crescentic glomerulonephritis (Figures 3 and 4). He underwent debridement of his bilateral foot ulcers and received an additional dose of rituximab. Following the second dose of rituximab, the oral ulcers improved. Toward the end of his hospitalization, his hemoglobin decreased and he was noted to have blood streaked sputum. Repeat CT chest was ordered and was consistent with diffuse alveolar hemorrhage (Figure 5). Unfortunately, he left prior to pulmonology evaluation. He was called and encouraged to come back to the hospital but declined and ultimately sought further medical attention at another hospital where he was ultimately admitted to the intensive care unit (ICU). Nephrology was consulted and began plasmapheresis for a total of 7 treatments. He was given a one-time dose of cyclophosphamide on initial presentation and then an additional dose of rituximab following plasmapheresis. He gradually improved and was discharged home 8 days after admission.

## 3. Discussion

Granulomatosis with polyangiitis was first described in 1936 by Wegener [10]. GPA was defined by the 2012 Chapel Hill consensus as necrotizing inflammatory vasculitis affecting mainly the small to medium vessels of the upper and lower respiratory tract [11]. Gubbels et al. reported that GPA affects a wide age range with a mean age of diagnosis of 40 years [12]. GPA can affect all racial groups but is seen predominantly among Caucasian people [13]. The typical diagnostic criteria were first described by Goldman and Churg in 1954. This includes upper and lower respiratory tract necrotizing granulomas, glomerulonephritis, and systemic vasculitis [14]. The American College of Rheumatology established criteria for GPA in 1990 which include abnormal urinary sediment, chest radiograph abnormalities, oral ulcers or nasal discharge, and biopsy showing granulomatous inflammation [15]. The earliest symptoms of the disease are often important clues that guide toward the diagnosis. Kihiczak published a case report in 1994 describing a 36-year-old woman who was diagnosed with sarcoidosis after having conjunctival and nasal mucosal biopsy. Later on, after a prolonged course of symptoms, she was ultimately properly diagnosed with atypical GPA with granulomatous ulcers [16]. Figarella also described a patient who had recurrent nodular lesions on the legs and biopsy showed nonspecific inflammation. Ten years later, pulmonary symptoms developed and a repeat skin biopsy revealed granulomatous vasculitis as the underlying cause for the cutaneous and pulmonary disease [17]. While skin lesions occur in 14–50% of patients with GPA, there are no specific cutaneous symptoms for the disease. Lesions can include palpable purpura, skin nodules, skin ulcers, and livedo reticularis. Palpable purpura can be considered as the most common skin manifestation with a prevalence of 35% among patients with GPA and 74% among dermatological findings [9]. A very rare cutaneous manifestation is pyoderma gangrenosum, defined by skin necrosis and deep ulcers. Kędzierska et al. published a treatment-resistant case that was treated with dapsone and steroids [18]. Sinovich and Snow also presented a case of a patient with right infra-auricular noduloulcerative lesion that progressed to a periauricular pyoderma gangrenosum that was completely resolved with steroids and azathioprine therapy [19]. Duna et al. reported that 6–13% of patients with GPA have oral lesions with 2% only have it as a presenting feature [20]. This was one of our patient's presenting complaints making this case somewhat unusual. The combination of “strawberry gingivitis” with a biopsy showing pseudoepitheliomatous hyperplasia, microabscesses, and multinucleated giant cells with severe systemic symptoms can be diagnostic for GPA [21]. There have been few reports showing that GPA can affect parotid, submandibular, and sublingual salivary glands [22]. Tongue necrosis is a rare oral symptom with only two previously reported cases. The first one was by Bachmeyer et al. who published a case of necrotic lingual ulceration that was resolved with immunosuppressive therapy [23]. The second was by Rodgers et al. with a case of bilateral tongue infarction with a severe and rapidly progressive form of GPA [24]. Early diagnosis is important. The aim of treating GPA early on is to prevent the morbidity and mortality associated with the disease as the average life expectancy is only 5 months for a patient without treatment with a 1-year survival rate <20% [25]. Treatment of GPA is multidisciplinary between different subspecialties in order to treat the different organs that are involved in the disease process. Treatment with corticosteroid alone was found to double the life expectancy to about 12 months with a 1-year survival of 34% [26]. Treatment with cyclophosphamide and corticosteroid therapy has resulted in remission and increased survival rate [27]. Azathioprine, while less effective than cyclophosphamide, has shown some success and should be considered in patients with adverse effects to cyclophosphamide or when patients have fertility concerns [28]. Methotrexate, used in limited GPA, is less likely to achieve and sustain remission [29]. Rituximab is a monoclonal antibody against CD20 antigen on B cells and may be important in prevention of disease relapse [30].

In conclusion, GPA should be considered in the differential among patients who present with skin and oral ulcers compatible with pyoderma gangrenosum that develop worsening of renal indices. Due to the morbidity and mortality associated with the condition, early diagnosis is important. A multidisciplinary approach with close communication between a renal team and primary care physician team is important to achieve the best outcomes. This role of the sickle cell trait in this patient is undefined, but this combination of gender, race, and presenting symptoms in GPA is extremely unusual.

## Data Availability

All data generated or analyzed in this study are included within this article. Access to data is possible with permission from the responsible author.
